# National Patterns of Risk-Standardized Mortality and Readmission After Hospitalization for Acute Myocardial Infarction, Heart Failure, and Pneumonia: Update on Publicly Reported Outcomes Measures Based on the 2013 Release

**DOI:** 10.1007/s11606-014-2862-5

**Published:** 2014-05-14

**Authors:** Lisa G. Suter, Shu-Xia Li, Jacqueline N. Grady, Zhenqiu Lin, Yongfei Wang, Kanchana R. Bhat, Dima Turkmani, Steven B. Spivack, Peter K. Lindenauer, Angela R. Merrill, Elizabeth E. Drye, Harlan M. Krumholz, Susannah M. Bernheim

**Affiliations:** 1 Yale New Haven Health Services Corporation/Center for Outcomes Research and Evaluation, 1 Church Street, Suite 200, New Haven, CT 06510 USA; 2 Section of Rheumatology, Yale School of Medicine, New Haven, CT USA; 3 Section of Cardiovascular Medicine, Yale University School of Medicine, New Haven, CT USA; 4 Taybah for Healthcare Consulting, Inc., Plano, TX USA; 5 Baystate Medical Center, Springfield, MA USA; 6 Tufts University School of Medicine, Boston, MA USA; 7 Mathematica Policy Research, Inc., Cambridge, MA USA; 8 Section of General Internal Medicine, Yale University School of Medicine, New Haven, CT USA

**Keywords:** myocardial infarction, heart failure, pneumonia, performance measurement, outcome measures, mortality, readmission

## Abstract

**BACKGROUND:**

The Centers for Medicare & Medicaid Services publicly reports risk-standardized mortality rates (RSMRs) within 30-days of admission and, in 2013, risk-standardized *unplanned* readmission rates (RSRRs) within 30-days of discharge for patients hospitalized with acute myocardial infarction (AMI), heart failure (HF), and pneumonia. Current publicly reported data do not focus on variation in national results or annual changes.

**OBJECTIVE:**

Describe U.S. hospital performance on AMI, HF, and pneumonia mortality and updated readmission measures to provide perspective on national performance variation.

**DESIGN:**

To identify recent changes and variation in national hospital-level mortality and readmission for AMI, HF, and pneumonia, we performed cross-sectional panel analyses of national hospital performance on publicly reported measures.

**PARTICIPANTS:**

Fee-for-service Medicare and Veterans Health Administration beneficiaries, 65 years or older, hospitalized with principal discharge diagnoses of AMI, HF, or pneumonia between July 2009 and June 2012. RSMRs/RSRRs were calculated using hierarchical logistic models risk-adjusted for age, sex, comorbidities, and patients’ clustering among hospitals.

**Results:**

Median (range) RSMRs for AMI, HF, and pneumonia were 15.1% (9.4–21.0%), 11.3% (6.4–17.9%), and 11.4% (6.5–24.5%), respectively. Median (range) RSRRs for AMI, HF, and pneumonia were 18.2% (14.4–24.3%), 22.9% (17.1–30.7%), and 17.5% (13.6–24.0%), respectively. Median RSMRs declined for AMI (15.5% in 2009–2010, 15.4% in 2010–2011, 14.7% in 2011–2012) and remained similar for HF (11.5% in 2009–2010, 11.9% in 2010–2011, 11.7% in 2011–2012) and pneumonia (11.8% in 2009–2010, 11.9% in 2010–2011, 11.6% in 2011–2012). Median hospital-level RSRRs declined: AMI (18.5% in 2009–2010, 18.5% in 2010–2011, 17.7% in 2011–2012), HF (23.3% in 2009–2010, 23.1% in 2010–2011, 22.5% in 2011–2012), and pneumonia (17.7% in 2009–2010, 17.6% in 2010–2011, 17.3% in 2011–2012).

**Conclusions:**

We report the first national unplanned readmission results demonstrating declining rates for all three conditions between 2009–2012. Simultaneously, AMI mortality continued to decline, pneumonia mortality was stable, and HF mortality experienced a small increase.

**Electronic supplementary material:**

The online version of this article (doi:10.1007/s11606-014-2862-5) contains supplementary material, which is available to authorized users.

## INTRODUCTION

The Centers for Medicare & Medicaid Services (CMS) began reporting hospital-specific 30-day risk-standardized mortality rates (RSMRs) for patients hospitalized with acute myocardial infarction (AMI) and heart failure (HF) in June 2007, and for patients hospitalized with pneumonia in 2008 through the Hospital Inpatient Quality Reporting (IQR) Program (formerly known as the Reporting Hospital Quality Data for Annual Payment Update program). In 2009, CMS also began reporting hospital-specific 30-day risk-standardized readmission rates (RSRRs) for all three conditions.^1^–^14^

Patients and other stakeholders can compare individual hospitals’ performance on these six quality measures on CMS’s *Hospital Compare* website.^15^ However, this resource does not provide information on the distribution of hospital performance for the nation as a whole, nor does it examine changes within the 3-year publicly reported measurement period. Although similar information was published in 2010,^16^,^17^ these measures are updated with new data each year and there has been no peer-reviewed summary since CMS began publicly reporting readmission rates. Furthermore, in 2011, CMS expanded national reporting to include Veterans Health Administration (VA) hospitals, and in 2013, updated the readmission measures to include only unplanned readmissions as measure outcomes.

We aim to describe the performance of U.S. hospitals on mortality and readmission measures following hospitalizations for AMI, HF, and pneumonia. Specifically, we summarize the national distribution of variation in hospital performance on the publicly reported outcomes measures based on the most current data (July 2009 to June 2012) and quantify annual changes within the 3-year period reported on *Hospital Compare*.

## METHODS

### Study Sample

The cohort for the six measures consists of hospitalizations for all Medicare fee-for-service (FFS) and VA beneficiaries aged 65 years and older, discharged from non-federal or VA acute care hospitals and having a principal discharge diagnosis of AMI, HF, or pneumonia. Medicare FFS beneficiaries are included if they have been enrolled in Medicare for the 12 months before and including the date of the index admission to ensure a full year of data for risk adjustment. The index hospitalization is the admission being measured for the outcome and identified based on patients’ principal discharge diagnosis. Discharges included in the publicly reported measures in 2013 occurred during July 2009 through June 2012. Additional details on measure methodology are provided in the online Appendix and published elsewhere.^1^–^14^ For patients with multiple hospitalizations for the same condition during a single year, the mortality measures include one randomly selected hospitalization per year per eligible patient per condition. For readmission measures, patients must be discharged alive for inclusion and may have multiple index admissions per year, but only admissions after 30 days of discharge from an index admission are eligible to be included as additional index admissions.

### Data Sources

The data sources are CMS administrative (claims and enrollment) data and VA administrative data. Index hospitalizations and readmissions are identified from inpatient claims. Dates of death were ascertained from the CMS enrollment database and VA records. To identify patient risk factors, the measures use inpatient and outpatient claims from the year prior to the index admission and secondary diagnosis codes from the index admission. The online Appendix contains additional details on measure methodology.

### Outcomes

The mortality measures assess death from any cause, within 30 days of admission for the index hospitalization. The mortality outcome is attributed to the initial admitting hospital, regardless of subsequent transfers.

The readmission measures assess unplanned re-hospitalization to any acute care facility for any cause within 30 days of discharge from the index hospitalization. Readmissions to observation status or non-acute units such as rehabilitation are not included. Readmission is attributed to the hospital that ultimately discharges a patient to a non-acute care setting (e.g. home, skilled nursing facility). Planned readmissions, such as hospitalizations reflecting readmissions for elective chemotherapy or scheduled procedures, are not considered outcome events in these measures and are defined using a publicly vetted approach that is delineated in the supplemental online Appendix.

### Risk-Standardized Rates

Details of the risk models used are provided in the online Appendix. The measures calculate hospital-level risk-standardized mortality or readmission rates using the hierarchical logistic regression model currently used in the publicly reported outcome measures to account for clustering of patients within hospitals while risk-adjusting for differences in patient case-mix. Each model includes age, sex, selected clinical covariates, and a hospital-specific random intercept using 3-year rolling combined or separate annual data. Comorbidities from the index admission that could represent complications of care are not included in the risk adjustment unless they are also present in claims within 12 months prior to admission to avoid attenuating the measures’ ability to characterize hospital care quality. The hospital-level RSMRs and RSRRs are calculated as ratios of the number of “predicted” to the number of “expected” outcome events for each hospital, which are then multiplied by the national unadjusted outcome rate. For each hospital, the ratio numerator is the number of outcome events predicted by the hospital’s performance with its observed case-mix (using a hospital-specific estimated intercept term), and the denominator is the number of expected deaths or readmissions based on the nation’s performance using the hospital’s observed case-mix. We used 5,000 bootstrap iterations to estimate 95% confidence intervals and variance for RSRRs/RSMRs for public reporting. Further details are provided elsewhere.^9^–^13^

### Analyses

To describe national hospital variation in publicly reported RSMRs/RSRRs, we evaluated their corresponding descriptive statistics, such as mean, standard deviation, median, and ranges. Hospitals were further characterized as “better” (“worse”) than the national rate if their risk-standardized rate was lower (higher) than the national rate and 95% confidence intervals did not include the national rate; if the hospital’s 95% confidence intervals included the national rate, the hospital was characterized as “no different than” the national rate.

To report annual changes in outcomes, a 1-year (July 1 through June 30) RSMR or RSRR was calculated for each hospital, condition, and measure for July 2009 through June 2012. For patients with multiple eligible admissions within a 1-year period, we selected a single index admission for inclusion in the mortality analyses. We calculated the median hospital rate and inter-quartile range at each time point for each measure. We used a generalized estimating equation (GEE) model to test whether the mean annual RSMR/RSRR changed significantly over the three year reporting period. We used annual hospital RSMRs/RSRRs as dependent variables and the three 12-month time periods as independent variables, and accounted for the correlation among the same hospitals over time. We also conducted pairwise comparisons of mean RSRRs or RSMRs between different time periods.

We calculated the number of hospitals changing performance categories and absolute differences in hospital-level risk-standardized rates between the 2012 (prior year) and 2013 (current) public reporting periods. We report these changes as “improved” (“worse than” to “no different than” national rate, “no different than” to “better than” national rate, or “worse than” to “better than” national rate), “no change”, or “worsened” (“no different than” to “worse than” national rate, “better than” to “no different than” national rate, or “better than” to “worse than” national rate).

All analyses were done with SAS version 9.2 (SAS Institute Inc., Cary, NC). We created figures with R (version 2.11.1).^18^ This work was exempted by the Yale University Human Investigation Committee.

## RESULTS

### Cohorts and Volumes

The measures include data from more than 4,500 hospitals. Over the 3-year period, we included 511,404 admissions in the AMI mortality measure cohort, 1,042,203 for HF mortality, and 1,037,583 for pneumonia mortality (Table 1). The case volume for the AMI, HF mortality, and pneumonia measures declined by 4–8% (7,094, 28,007 and 14,247 patients, respectively) between the first and last year of the 2013 reporting period. For the readmission measures, there were 513,331 AMI, 1,262,826 HF, and 1,089,758 pneumonia discharges over the three-year period and even larger declines (9–15%) between the first and last year of the reporting period (Table 1).

**Table 1:** Study Sample Characteristics and Crude Outcome Rates

	Mortality			Readmission		
Characteristics	AMI	HF	Pneumonia	AMI	HF	Pneumonia
No. of Cases						
July 2009 – June 2012 (full reporting period)	511,404	1,042,203	1,037,583	513,331	1,262,826	1,089,758
July 2009 – June 2010	173,576	359,589	347,585	177,031	447,351	371,002
July 2010 – June 2011	171,346	351,032	356,660	176,195	434,083	382,700
July 2011 – June 2012	166,482	331,582	333,338	160,105	381,392	336,056
No. of Hospitals	4,564	4,777	4,817	4,464	4,786	4,833
Patient Age, years						
Median	79	82	81	79	81	80
25th percentile	72	75	74	72	74	74
75th percentile	86	87	87	85	87	86
Non-white, %	12.4	15.8	11.7	12.3	16.7	11.7
Eligible Hospital Cases^*^						
Median	40	117	150	30	137	158
25th percentile	10	42	68	7	48	71
75th percentile	156	308	297	148	370	310
Crude National Rates for 3-Year Combined Data Used in Public Reporting, %						
July 2009 – June 2012 (full reporting period)	15.2	11.7	11.9	18.3	23.0	17.6
Annual Crude National Rates, %						
July 2009 – June 2010	15.5	11.5	11.9	18.6	23.4	17.7
July 2010 – June 2011	15.4	11.9	12.0	18.5	23.2	17.7
July 2011 – June 2012	14.8	11.7	11.7	17.7	22.5	17.3

^*^Eligible Hospital Cases represent the number of index hospitalizations per hospital meeting criteria for inclusion in the measure cohort within the 3-year measurement period; for example, the median number of admissions per hospital included in the final Acute Myocardial Infarction (AMI) measure cohort was 40 hospitalizations

### Variation in Hospital-Specific Risk-Standardized Rates (Table 2 and Figs. 1–2)

**Table 2:** Risk-Standardized Outcome Rates Following Hospitalizations for AMI, HF, and Pneumonia

	Mortality			Readmission		
Outcome	AMI	HF	Pneumonia	AMI	HF	Pneumonia
Risk-Standardized Rates for 3-Year Combined Data Used in Public Reporting, %						
Distribution of weighted RSMRs/RSRRs^a^, July 2009 – June 2012 combined, %						
Mean (SD)	15.3 (1.2)	11.8 (1.5)	12.0 (1.8)	18.3 (0.9)	23.1 (1.7)	17.6 (1.4)
Minimum	9.4	6.4	6.5	14.4	17.1	13.6
1st percentile	11.5	7.8	7.8	15.5	18.4	14.6
5th percentile	12.6	8.8	8.8	16.5	20.0	15.4
10th percentile	13.3	9.4	9.4	17.1	20.7	15.8
25th percentile	14.3	10.3	10.3	17.8	21.8	16.6
Median	15.1	11.3	11.4	18.2	22.9	17.5
75th percentile	15.8	12.3	12.6	18.7	24.2	18.4
90th percentile	16.5	13.4	13.9	19.5	25.3	19.5
95th percentile	17.0	14.0	14.8	20.0	26.1	20.2
99th percentile	18.2	15.4	16.4	21.1	27.9	21.6
Maximum	21.0	17.9	24.5	24.3	30.7	24.0
Annual Risk-Standardized Rates, %						
Median Annual Hospital-Level RSMRs/RSRRs, %						
July 2009 – June 2010	15.5	11.5	11.8	18.5	23.3	17.7
July 2010 – June 2011	15.4	11.9	11.9	18.5	23.1	17.6
July 2011 – June 2012	14.7	11.7	11.6	17.7	22.5	17.3

RSMRs/RSRRs are weighted by inverse of variance of RSRR/RSMR calculated from bootstrapping; RSMR risk-standardized mortality ratio; RSRR risk-standardized readmission ratio

**Figure 1:**
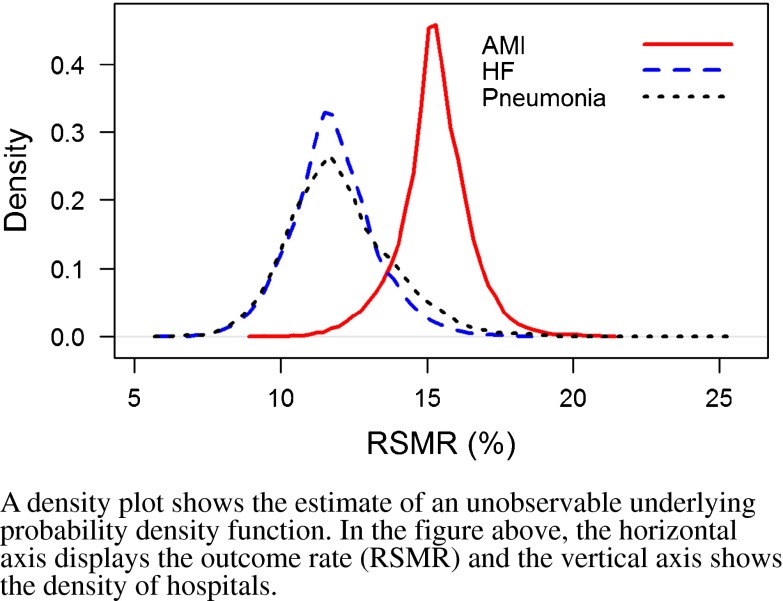
Density plot of the distributions of hospital-level risk-standardized mortality rates (RSMRs) following admission for acute myocardial infarction (AMI), heart failure (HF) and pneumonia, for the period 1 July 2009 through 30 June 2012.

**Figure 2:**
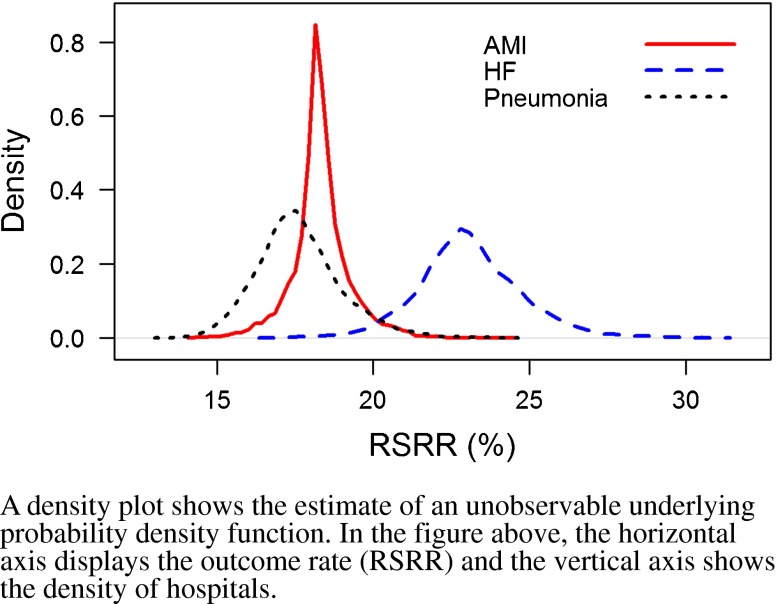
Density plot of the distributions of hospital-level risk-standardized readmission rates (RSRRs) following admission for acute myocardial infarction (AMI), heart failure (HF) and pneumonia, for the period 1 July 2009 through 30 June 2012.

For the mortality measures, there was an absolute difference of 4.4%, 5.2%, and 6.0% between the median RSMRs for the 5^th^ and 95^th^ percentiles of hospitals for AMI, HF, and pneumonia, respectively. For the readmission measures, the absolute difference between the 5th and 95th percentiles of hospital RSRRs was 3.5%, 6.1%, and 4.8% for AMI, HF, and pneumonia, respectively.

### Changes in Hospital Performance (Tables 2 and 3)

**Table 3:** Number of Hospitals With a Change in Performance Categorization (Current Reporting Period 1 July 2009 to 30 June 2012 Compared to Prior Year Period 1 July 2008 to 30 June 2011)

	Mortality			Readmission		
Change in Categorization	AMI (*N* = 2,587)	HF (*N* = 3,929)	Pneumonia (*N* = 4,313)	AMI (*N* = 2,283)	HF (*N* = 4,021)	Pneumonia (*N* = 4,334)
No. Improved						
Worse Than to No Different Than National Rate	17	54	95	28	63	53
No Different Than to Better Than National Rate	32	62	76	9	56	24
Worse Than to Better Than National Rate	0	0	0	0	0	0
No. with No Change						
Worse Than National Rate	6	63	121	13	97	68
No Different Than National Rate	2,448	3,482	3,737	2187	3,652	4,092
Better Than National Rate	45	116	124	14	64	13
No. Worsened						
No Different Than to Worse Than National Rate	13	73	99	16	59	24
Better Than to No Different Than National Rate	26	79	61	10	30	20
Better Than to Worse Than National Rate	0	0	0	0	0	0

Excludes hospitals with fewer than 25 cases during the reporting period as well as critical access hospitals and other hospitals not participating in the Hospital Inpatient Quality Reporting program who requested that CMS suppress their results: AMI Acute Myocardial Infarction; HF Heart Failure; Hospitals were characterized as “better” (“worse”) than the national rate if their risk-standardized rate was lower (higher) than the national rate and 95% confidence intervals did not include the national rate; if the hospital’s 95% confidence intervals included the national rate, the hospital was characterized as “no different than” the national rate

The annual hospital-level 30-day RSMRs revealed a decline of 0.8% in the median (Table 2) and mean rates for AMI (data not shown, *p* < 0.001 for trend). During the same period, the median and mean annual hospital-level 30-day RSMR for HF increased by an absolute 0.2% (*p* < 0.001 for trend between 2009–2010 and 2011–2012, first and last years of current reporting period) and decreased for pneumonia by an absolute 0.2% (*p* < 0.001 for trend between 2009–2010 and 2011–2012, the first and last years of current reporting period).

There has been a statistically and clinically significant decrease in the annual mean RSRRs for all three measures (absolute decreases of 0.8% for AMI, 0.8% for HF and 0.4% for pneumonia, all *p* < 0.001, Table 2) between the first and last year of the 2013 reporting period.

Between the 2012 reporting period and the 2013 reporting period, no hospital moved from better to worse than the national rate or vice versa (Table 3). Across the six measures, 93–97% of hospitals remained in the same performance category; between 1.6% (AMI readmission) and 4.0% (pneumonia mortality) of hospitals improved categories; and between 1.0% (pneumonia readmission) to 3.9% (pneumonia mortality) of hospitals moved to a lower performance category. Median RSMRs for hospitals performing “better than the national rate” were 7.7%, 6.4%, and 7.0% lower for AMI, HF, and pneumonia, respectively, than median RSMRs for those hospitals performing “worse than the national rate.” Median RSRRs were 6.4%, 7.6%, and 6.7% lower for “better than national rate” compared to “worse than national rate” hospitals for the AMI, HF, and pneumonia readmission measures, respectively.

## DISCUSSION

Between July 2009 and June 2012, the current 3-year measurement period for CMS’s public reporting of hospital performance, our results demonstrate improvement in all annual risk-standardized outcome rates for patients admitted with AMI, HF, and pneumonia, except an increase of 0.2% absolute percentage points in HF mortality. Despite this improvement, in half of U.S. hospitals, one in seven patients admitted for AMI will die within 30 days of admission and one in four patients admitted with HF will be readmitted with an unplanned readmission within 30 days of discharge. Given the complexity of improving outcomes, improvement in national rates is likely to be slow, but these results allow an assessment of the trajectory of outcomes within the 3 years included in current publicly-reported results, showing that mortality following admission for AMI continues to decline and unplanned readmission rates for all three conditions indicate early signs of improvement. However, HF mortality rose slightly over the reporting period and pneumonia mortality did not demonstrate the marked declines shown prior to 2005.^19^ These results reflect outcomes after the start of public reporting and inclusion of these measures in CMS’s pay-for-performance programs.^20^ In addition, the readmission rates now focus solely on unplanned readmissions.

The measures continue to demonstrate wide performance variation, with outcome rates for high performing hospitals (10^th^ percentile) at least three absolute percentage points lower than those for poor performing hospitals (90^th^ percentile) for five of six measures; the AMI readmission measure demonstrates 2.4 absolute percentage points between top and bottom deciles. Documentation of this variation provides evidence of continued room for improvement in care quality. The publicly reported measures are designed to benchmark an individual hospital against how an average hospital performs when caring for a similar case-mix. The goal of public reporting is not to reduce mortality or readmission to zero, but rather to bring as many hospitals as possible to the performance standards set by the nation’s highest performing hospitals.

Although cross-sectional and including only the 3-year current measurement period, these data are the first indication that measure-specific readmission rates are declining and support evidence published by Gerhardt, et. al in 2013 that all-condition readmission rates are declining.^21^ Unlike that study as well as previous hospital-level results reported on CMS’s *Hospital Compare*, our findings indicate declining rates of *unplanned* readmissions after hospitalization for AMI, HF, and pneumonia. These are the first data to exclude planned readmissions from the measures’ outcome and therefore it is inappropriate to compare them directly to prior publicly reported results. However, by excluding planned readmissions, such as elective surgeries or planned chemotherapy, these results better assess hospital care quality. Using a uniform definition of unplanned readmission, median annual RSRRs for AMI and HF decreased by nearly a full (absolute) percentage point within the last 3 years of available data, compared to year-to-year changes in annual median RSRRs ≤ 0.3 absolute percentage points between 2007 and 2010.^22^,^23^ While some year-to-year variation in rates cannot be excluded, given the magnitude of recent changes compared to recent historic data, these improvements are unlikely to be random variation. If all hospitals with RSRRs in excess of the median value had improved to the median hospital RSRR, 5,072 AMI, 11,839 HF, and 14,494 pneumonia unplanned readmissions would have been averted during the 2013 public reporting period.

One concern expressed by critics of examining hospital readmissions as a measure of hospital quality is that focus on readmissions will divert attention from other important quality improvement efforts and/or result in unintended consequences.^24^ The most serious concern is that efforts to reduce readmissions may lead hospitals to avoid re-hospitalizing patients who need more intensive care, thus leading to increased mortality rates in the post-discharge period. With the exception of HF, which demonstrated a 0.2% absolute percentage point increase in median hospital RSMRs between the first and last year of the current 3-year reporting period, AMI and pneumonia mortality rates fell or were stable simultaneous to declining readmission rates. While this does not exclude a relationship between hospital performance on mortality and readmission measures, this finding is reassuring and supports earlier data that good performance on readmission measures does not necessarily occur at the expense of performance on the mortality measures or vice versa.^25^ Gorodeski et al. demonstrated that, at higher readmission rates, lower HF mortality was associated with higher readmission; however, this relationship did not exist for hospitals with the lowest readmission rates, where mortality performance remained flat,^26^ and the study did not examine outcomes for other conditions that have been studied previously and found to have little correlation.^25^ There is evidence that patients admitted now are sicker, as each measure shows small increases in the frequency of several risk covariates, such as cardiorespiratory failure and shock, over the measurement period (online Appendix), supporting that the patient case mix may be increasing in severity overtime. Despite this, only HF mortality showed an overall increase in mortality, and in fact, there was a decrease in HF mortality between the second and third years of the reporting period. These data merit further consideration, but reinforce the benefit of measuring and reducing both mortality and readmission rates simultaneously to prevent unintended consequences of measurement and reporting. Further, these measures supplement data captured by existing publicly reported process measures that examine and incentivize guideline-concordant care, but often correlate poorly with actual patient outcomes.^27^

There are several limitations to consider in interpreting these results. First, administrative claims carry less face validity for adequate case-mix adjustment compared to clinical data. The risk models used in these measures have been validated against and demonstrate similar performance assessments to models based on medical record data.^9^–^13^ Second, reported rates only reflect the experience of Medicare FFS and VA patients ages ≥ 65 and are not necessarily generalizable to other populations. Third, Medicare and VA data are combined prior to fitting the models, allowing between hospital means and variances to be different for the two systems. We did not explore whether VA hospitals represented a disproportionate number of improving hospitals; however, VA hospitals contribute less than 3% of measured patients and therefore the rates are largely driven by outcomes for index admissions to non-federal hospitals. Fourth, although we have used a robust risk-adjustment approach endorsed by statistical experts,^28^ there may be other sources of variation contributing to the differences among hospital RSMRs and RSRRs, including differences in coding practices that could impact either risk adjustment or cohort definitions. This has been identified as a concern for the pneumonia measures, with recent evidence suggesting that more seriously ill pneumonia patients may be coded with respiratory failure or sepsis and thus may not be included in measurement.^29^ Further, the measures do not adjust for code status or do not resuscitate orders, as these are inconsistently applied by clinicians and not captured in claims. However, the mortality measures exclude patients enrolled in hospice at any time during the 12-months prior to admission or who enrolled in hospice on the first day of admission, as the goals of care for such patients likely differ from patients not participating in hospice. These measures have been previously validated using medical record-based models, minimizing the likelihood that coding differences are the main source of variation for all measures. Variation in the propensity to admit patients or in care intensity may also contribute to performance differences.

Examination of 2009–2012 data reveals variation in the quality of care provided to patients with AMI, HF, and pneumonia. Our study summarizes unplanned readmission rates nationally and signals potential early declines in readmission rates. The 2013 publicly reported measures update suggests payment incentive programs may be influencing hospital readmission performance without a concomitant increase in mortality, at least for AMI and pneumonia patients, and supports the need for simultaneously measuring mortality and readmission to encourage better quality across all aspects of care and monitor for unintended consequences.

## Electronic supplementary material

Below is the link to the electronic supplementary material.

ESM 1 (DOCX 382 kb)
