# Supplementary material for: National Patterns of Risk-Standardized Mortality and Readmission After Hospitalization for Acute Myocardial Infarction, Heart Failure, and Pneumonia: Update on Publicly Reported Outcomes Measures Based on the 2013 Release
Source: J Gen Intern Med. 2014 May 14;29(10):1333–40. doi: 10.1007/s11606-014-2862-5 (PMC4175654; doi:10.1007/s11606-014-2862-5)
Supplement: Supplementary file 1 — (DOCX 382 kb) [file 11606_2014_2862_MOESM1_ESM.docx]

**Appendix:** **Supplemental Material**

**for**

**National Patterns of Risk-Standardized Mortality and Readmission after Hospitalization for Acute Myocardial Infarction, Heart Failure and Pneumonia: Update on Publicly Reported Outcomes Measures Based on the 2013 Release**

**Measure Methodology**

**AMI, Heart Failure, and Pneumonia Mortality and Readmission Measure Cohorts**

The acute myocardial infarction (AMI), heart failure, and pneumonia mortality and readmission measures include admissions for Medicare fee-for-service (FFS) and Veterans Health Administration (VA) hospital beneficiaries aged 65 years and older who were discharged from non-federal acute care hospitals or VA hospitals with a principal discharge diagnosis of AMI, heart failure, or pneumonia. Medicare FFS beneficiaries with an index admission to a non-federal hospital are included if they have been enrolled in Part A and Part B Medicare for the 12 months prior to and including the date of the index admission to ensure a full year of administrative data for risk adjustment. (This requirement is dropped for patients with an index admission to a VA hospital.) An index admission is the hospitalization considered for the mortality or readmission outcome. For the mortality measures only, for patients with more than one admission in a given year for a given condition, only one index admission for that condition is randomly selected for inclusion in the cohort.

The measures were developed using Medicare FFS administrative data but are designed for and have been tested for use in all-payer claims datasets.

**International Classification of Diseases, Ninth Revision, Clinical Modification (ICD-9-CM) Codes Defining AMI, Heart Failure, and Pneumonia**

The specific ICD-9-CM codes meeting the inclusion criteria for AMI, heart failure, and pneumonia are as follows:

For the AMI measure: 410.00, 410.01, 410.10, 410.11, 410.20, 410.21, 410.30, 410.31, 410.40, 410.41, 410.50, 410.51, 410.60, 410.61, 410.70, 410.71, 410.80, 410.81, 410.90, and 410.91

For the heart failure measure: 402.01, 402.11, 402.91, 404.01, 404.03, 404.11, 404.13, 404.91, 404.93, 428.0, 428.1, 428.20, 428.21, 428.22, 428.23, 428.30, 428.31, 428.32, 428.33, 428.40, 428.41, 428.42, 428.43, and 428.9

For the pneumonia measure: 480.0, 480.1, 480.2, 480.3, 480.8, 480.9, 481, 482.0, 482.1, 482.2, 482.30, 482.31, 482.32, 482.39, 482.40, 482.41, 482.42, 482.49, 482.81, 482.82, 482.83, 482.84, 482.89, 482.9, 483.0, 483.1, 483.8, 485, 486, 487.0, and 488.11

**Exclusion Criteria**

Mortality Measures

The AMI, heart failure, and pneumonia mortality measures exclude index admissions for patients:

- Discharged alive on the day of admission or the following day who were not transferred because it is unlikely they had a clinically significant diagnosis of AMI, heart failure, or pneumonia;
- Who were transferred from another acute care hospital or VA hospital (the acute episode is included in the measure but the death is attributed to the hospital where the patient was initially admitted rather than the hospital receiving the transferred patient);
- With inconsistent or unknown vital status or other unreliable data (for example, date of death precedes date of admission);
- Who were enrolled in the Medicare or VA Hospice programs any time in the 12 months prior to the index admission, including the first day of the index admission, as these patients were likely continuing to seek comfort measures only;
- Who were discharged against medical advice (AMA) because providers did not have the opportunity to deliver full care and prepare the patient for discharge; or
- Whose admission was not the first admission in the 30 days prior to a patient’s death. This exclusion criterion is applied after one admission per patient per year is randomly selected; thus, it is only applicable to the three-year combined data. Also, the exclusion only happens when two randomly-selected admissions occur during the transition months (June and July for data used in this report) and the patient subsequently dies. For example: a patient is admitted on June 18, 2010, readmitted on July 2, 2010, and dies on July 15, 2010. If both of these admissions are randomly selected for inclusion (one for the July 2009 – June 2010 time period and the other for the July 2010 – June 2011 time period), the July 2, 2010 admission will be excluded to avoid assigning the death to two admissions (one between July 2009 and June 2010, and one between July 2010 and June 2011).[^1^](#_ENREF_1)

For patients with more than one admission in a given year for a given condition, only one index admission for that condition is randomly selected for inclusion in the cohort.

Readmission Measures

The AMI, heart failure, and pneumonia readmission measures exclude index admissions for patients:

- With an in-hospital death;
- Without at least 30 days post-discharge enrollment in FFS Medicare because the 30-day readmission outcome cannot be assessed in this group. This exclusion applies only to patients who have index admissions in non-VA hospitals;
- Who were transferred to another acute care facility, as described further below, because the measure evaluates hospitalizations for patients discharged to non-acute care settings; or
- Who were discharged against medical advice (AMA), because providers did not have the opportunity to deliver full care and prepare the patient for discharge.

Readmissions within 30 days of discharge from an index admission will not be considered index admissions. Thus, no hospitalization will be counted as both a readmission and an index admission within the same measure. However, because the cohorts for the readmission measures are determined independently of each other, a readmission in one measure may qualify as an index admission in other CMS readmission measures.

An additional exclusion criterion for the AMI cohort is that patients admitted and then discharged on the same day are not included as an index admission because it is unlikely that these are clinically significant AMIs.[^2^](#_ENREF_2)

Additional information on the methodology of the measures, including how transfers are handled, the size of exclusions for each measurement period, and the rationale for the outcome ascertainment periods, are detailed in the annual Measure Methodology and Maintenance reports.[^1-16^](#_ENREF_1)

**AMI, Heart Failure, and Pneumonia Readmission Measure Outcome Definition**

To more broadly identify planned readmissions, CMS contracted with CORE to develop a planned readmission “algorithm” (a set of criteria) for classifying readmissions as planned using Medicare fee-for-service claims.[^17^](#_ENREF_17) The algorithm identifies admissions that are typically planned and may occur within 30 days of discharge from the hospital.

We based the planned readmission algorithm on three principles:

1. A few specific, limited types of care are always considered planned (obstetrical delivery, transplant surgery, maintenance chemotherapy, and rehabilitation);

2. Otherwise, a planned readmission is defined as a non-acute readmission for a scheduled procedure; and

3.  Admissions for acute illness or for complications of care are never planned.

In brief, the planned readmission algorithm uses a flow chart (Figure A1) and four tables of specific procedure categories and discharge diagnosis categories to classify readmissions as planned. The algorithm first checks if the readmission had a primary discharge diagnosis that is ALWAYS considered planned, or if the readmission had a procedure for which readmissions are always considered planned. If the readmission does not qualify as always planned, the algorithm then checks if the readmission had a procedure that is considered potentially planned. If not, the readmission is considered unplanned. If the readmission does have a potentially planned procedure, however, the algorithm will do the final check for primary discharge diagnoses that are considered acute. If the potentially planned readmission has an acute primary discharge diagnosis, the readmission is considered unplanned. If the potentially planned readmission does not have an acute primary discharge diagnosis, however, the readmission is considered planned.

The planned readmission algorithm was developed in a hospital-wide cohort of patients and has had extensive public input. Clinicians in our internal working group reviewed the full list of Agency for Healthcare Research and Quality Procedure Clinical Classification Software codes and identified procedure categories that are commonly planned. The initial detailed list of planned readmissions and acute diagnoses was posted as part of two public comment periods for the hospital-wide readmission measure. Subsequently, the algorithm was reviewed by 27 surgical subspecialists nominated by their specialty societies and by hospitals participating in a national dry run (confidential reporting) of two CMS readmission measures (the hospital-wide and total hip or knee arthroplasty readmission measures). In addition, the algorithm has been posted for public comment during National Quality Forum reviews of multiple CMS readmission measures. CMS has revised the algorithm in response to these reviews and, as of December 2013, is currently using Version 2.1, adapted as appropriate for condition-specific and procedure-specific patient cohorts, in all of its measures.

Figure A1. Planned Readmission Algorithm Version 2.1 – Flowchart


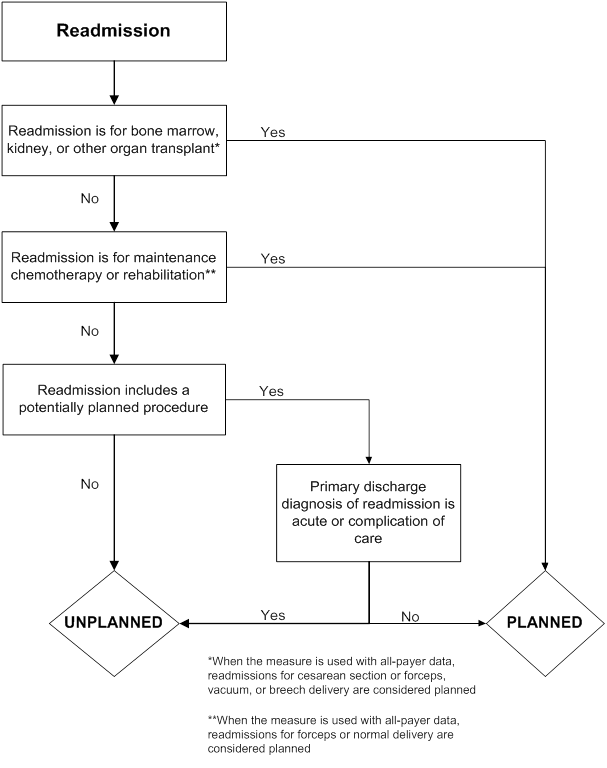


**AMI, Heart Failure, and Pneumonia Mortality and Readmission Measure Risk Models**

Tables A1-A7 below provide A) the list of conditions that may represent adverse outcomes of the care received during an index admission and are therefore excluded from the measure risk models unless they are also present in the 12 months prior to the index admission, and B) the list of risk covariates included in the measure risk models and their frequency over the three-year measurement period. For the readmission measures, where the discharging hospital is held responsible for readmissions, if a patient is transferred between hospitals, the secondary diagnosis codes from the transferring hospital *are* included in the risk model. In this way, complications of care occurring at the transferring hospital are included in the risk adjustment of the likelihood of readmission from the discharging hospital. In addition, the risk models do not account for either the urgency of the admission or whether the patient has a do not resuscitate (DNR) order in place. The 30-day mortality measures exclude patients who were enrolled in the Medicare hospice program or in VA hospice programs at any time during the 12 months prior to the index admission or who enrolled in the hospice program on the first day of the index admission. Neither the urgency of the admission nor documentation of DNR status is accurately captured in administrative data. In addition, these variables, were they available, may reflect differences in care practices across hospitals that would potentially mask performance differences if included in the risk models. The risk models have been validated and shown to perform well compared to clinical risk models that employ medical record data.[^3^](#_ENREF_3)^,^[^4^](#_ENREF_4)^,^[^6-8^](#_ENREF_6)

Table A1. Conditions That May Represent Adverse Outcomes of Care Received During Index Admission and Are Excluded from Risk Models Unless Present in the 12 Months Prior to the Index Admission

| **CC #** | **Description** |
| --- | --- |
| 2 | Septicemia/Shock |
| 6 | Other Infectious Diseases |
| 17 | Diabetes with Acute Complications |
| 23 | Disorders of Fluid/Electrolyte/Acid-Base Balance |
| 28 | Acute Liver Failure/Disease |
| 31 | Intestinal Obstruction/Perforation |
| 34 | Peptic Ulcer, Hemorrhage, Other Specified Gastrointestinal Disorders |
| 46 | Coagulation Defects and Other Specified Hematological Disorders |
| 48 | Delirium and Encephalopathy |
| 75 | Coma, Brain Compression/Anoxic Damage |
| 77 | Respirator Dependence/Tracheostomy Status |
| 78 | Respiratory Arrest |
| 79 | Cardio-Respiratory Failure and Shock |
| 80 | Congestive Heart Failure |
| 81 | Acute Myocardial Infarction |
| 82 | Unstable Angina and Other Acute Ischemic Heart Disease |
| 92 | Specified Heart Arrhythmias |
| 93 | Other Heart Rhythm and Conduction Disorders |
| 95 | Cerebral Hemorrhage |
| 96 | Ischemic or Unspecified Stroke |
| 97 | Precerebral Arterial Occlusion and Transient Cerebral Ischemia |
| 100 | Hemiplegia/Hemiparesis |
| 101 | Cerebral Palsy and Other Paralytic Syndromes |
| 102 | Speech, Language, Cognitive, Perceptual Deficits |
| 104 | Vascular Disease with Complications |
| 105 | Vascular Disease |
| 106 | Other Circulatory Disease |
| 111 | Aspiration and Specified Bacterial Pneumonias |
| 112 | Pneumococcal Pneumonia, Empyema, Lung Abscess |
| 114 | Pleural Effusion/Pneumothorax |
| 130 | Dialysis Status |
| 131 | Renal Failure |
| 132 | Nephritis |
| 133 | Urinary Obstruction and Retention |
| 135 | Urinary Tract Infection |
| 148 | Decubitus Ulcer of Skin |
| 152 | Cellulitis, Local Skin Infection |
| 154 | Severe Head Injury |
| 155 | Major Head Injury |
| 156 | Concussion or Unspecified Head Injury |
| 158 | Hip Fracture/Dislocation |
| 159 | Major Fracture, Except of Skull, Vertebrae, or Hip |
| 163 | Poisonings and Allergic Reactions |
| 164 | Major Complications of Medical Care and Trauma |
| 165 | Other Complications of Medical Care |
| 174 | Major Organ Transplant Status |
| 175 | Other Organ Transplant/Replacement |
| 177 | Amputation Status, Lower Limb/Amputation Complications |
| 178 | Amputation Status, Upper Limb |

Data on the frequency of risk variables for the AMI, HF, and pneumonia mortality and readmission models listed below is based upon calendar year data from July 2009 through June 2012

Table A2– Frequency of AMI Mortality Model Variables over Different Time Periods

| **Variable** | **07/2009-06/2010** | **07/2010-06/2011** | **07/2011-06/2012** | **07/2009-06/2012** |
| --- | --- | --- | --- | --- |
| Total N | 173,576 | 171,355 | 166,494 | 511,404 |
| Observed mortality rate | 15.5 | 15.4 | 14.8 | 15.2 |
| Mean Age (SD) | 79.2 (8.3) | 79.3 (8.3) | 79.1 (8.3) | 79.2 (8.3) |
| Male | 50.8 | 50.9 | 51.7 | 51.1 |
| History of PTCA | 8.0 | 11.8 | 15.8 | 11.8 |
| History of CABG | 5.8 | 8.9 | 11.9 | 8.8 |
| Congestive heart failure (CC 80) | 31.0 | 31.1 | 30.9 | 31.0 |
| Acute myocardial infarction (CC 81) | 13.5 | 14.0 | 13.5 | 13.7 |
| Other acute/subacute forms of ischemic heart disease (CC 82) | 13.3 | 13.2 | 13.4 | 13.3 |
| Anterior myocardial infarction (ICD-9 codes 410.00-410.19) | 9.1 | 8.6 | 8.3 | 8.7 |
| Other location of myocardial infarction (ICD-9 codes 410.20-410.69) | 12.8 | 12.3 | 12.2 | 12.5 |
| Chronic atherosclerosis (CC 83, 84) | 77.6 | 80.9 | 84.5 | 81.0 |
| Cardio-respiratory failure and shock (CC 79) | 9.4 | 9.9 | 10.3 | 9.9 |
| Valvular and rheumatic heart disease (CC 86) | 26.1 | 28.8 | 31.5 | 28.8 |
| Hypertension (CC 89, 91) | 84.4 | 86.6 | 88.9 | 86.6 |
| Stroke (CC 95-96) | 7.9 | 7.7 | 7.5 | 7.7 |
| Cerebrovascular disease (CC 97-99, 103) | 19.8 | 20.3 | 21.0 | 20.4 |
| Renal failure (CC 131) | 22.9 | 24.6 | 26.2 | 24.5 |
| Chronic obstructive pulmonary disease (COPD) (CC 108) | 28.1 | 29.6 | 30.9 | 29.5 |
| Pneumonia (CC 111-113) | 23.8 | 24.0 | 23.7 | 23.8 |
| Diabetes mellitus (DM) or DM complications (CC 15-20, 120) | 43.4 | 45.0 | 46.5 | 44.9 |
| Protein-calorie malnutrition (CC 21) | 5.4 | 6.2 | 6.6 | 6.0 |
| Dementia or senility (CC 49, 50) | 18.3 | 19.7 | 20.8 | 19.6 |
| Hemiplegia, paraplegia, paralysis,  functional disability (CC 67-69, 100-102, 177, 178) | 5.9 | 6.1 | 6.5 | 6.2 |
| Peripheral vascular disease (CC 104, 105) | 26.4 | 26.7 | 27.7 | 26.9 |
| Metastatic cancer, acute leukemia and other major cancers (CC 7, 8) | 3.9 | 3.8 | 4.0 | 3.9 |
| Trauma in last year (CC 154-156, 158-162) | 30.4 | 31.2 | 31.7 | 31.1 |
| Major psychiatric disorders (CC 54-56) | 7.0 | 7.6 | 8.2 | 7.6 |
| Chronic liver disease (CC 25-27) | 1.1 | 1.2 | 1.4 | 1.2 |

Table A3– Frequency of HF Mortality Model Variables over Different Time Periods

| **Variable** | **07/2009-06/2010** | **07/2010-06/2011** | **07/2011-06/2012** | **07/2009-06/2012** |
| --- | --- | --- | --- | --- |
| Total N | 359,589 | 351,109 | 331,662 | 1,042,203 |
| Observed mortality rate | 11.5 | 11.9 | 11.7 | 11.7 |
| Mean Age (SD) | 81.1 (8.1) | 81.2 (8.2) | 81.2 (8.2) | 81.1 (8.2) |
| Male | 45.0 | 45.0 | 45.5 | 45.2 |
| History of PTCA | 6.0 | 9.4 | 12.7 | 9.3 |
| History of CABG | 8.4 | 13.9 | 18.8 | 13.6 |
| Congestive heart failure (CC 80) | 74.1 | 74.4 | 74.8 | 74.4 |
| Acute myocardial infarction (CC 81) | 9.7 | 9.7 | 9.7 | 9.7 |
| Other acute/subacute forms of ischemic heart disease (CC 82) | 12.4 | 12.2 | 12.2 | 12.3 |
| Chronic atherosclerosis (CC 83, 84) | 69.9 | 71.5 | 73.5 | 71.6 |
| Cardio-respiratory failure and shock (CC 79) | 22.3 | 23.7 | 25.6 | 23.8 |
| Valvular and rheumatic heart disease (CC 86) | 45.1 | 48.5 | 52.6 | 48.6 |
| Hypertension (CC 89, 91) | 89.5 | 91.5 | 93.3 | 91.4 |
| Stroke (CC 95, 96) | 10.0 | 9.8 | 9.7 | 9.8 |
| Renal failure (CC 131) | 43.2 | 46.1 | 49.0 | 46.0 |
| COPD (CC 108) | 45.5 | 47.1 | 48.7 | 47.0 |
| Pneumonia (CC 111-113) | 43.7 | 44.4 | 44.9 | 44.3 |
| Diabetes mellitus (DM) and DM complications (CC 15-20, 120) | 51.2 | 52.5 | 53.9 | 52.5 |
| Protein-calorie malnutrition (CC 21) | 8.4 | 9.4 | 10.3 | 9.3 |
| Dementia and senility (CC 49, 50) | 21.7 | 23.4 | 25.5 | 23.5 |
| Hemiplegia, paraplegia, paralysis, functional disability (CC 67-69, 100102, 177, 178) | 7.4 | 7.8 | 8.7 | 8.0 |
| Peripheral vascular disease (CC 104, 105) | 35.5 | 36.4 | 38.5 | 36.7 |
| Metastatic cancer, acute leukemia, and other severe cancers (CC 7, 8) | 4.3 | 4.3 | 4.4 | 4.3 |
| Trauma in last year (CC 154-156, 158-162) | 38.1 | 39.4 | 40.3 | 39.2 |
| Major psychiatric disorders (CC 54-56) | 9.5 | 10.2 | 10.9 | 10.2 |
| Chronic liver disease (CC 25-27) | 2.2 | 2.5 | 3.0 | 2.6 |

Table A4– Frequency of Pneumonia Mortality Model Variables over Different Time Periods

| **Variable** | **07/2009-06/2010** | **07/2010-06/2011** | **07/2011-06/2012** | **07/2009-06/2012** |
| --- | --- | --- | --- | --- |
| Total N | 347,585 | 356,694 | 333,370 | 1,037,583 |
| Observed mortality rate | 11.9 | 12.0 | 11.7 | 11.9 |
| Mean Age (SD) | 80.3 (8.2) | 80.5 (8.3) | 80.4 (8.3) | 80.4 (8.3) |
| Male | 45.9 | 46.0 | 46.3 | 46.1 |
| History of PTCA | 3.2 | 5.3 | 7.0 | 5.2 |
| History of CABG | 4.1 | 7.1 | 9.3 | 6.8 |
| Congestive heart failure (CC 80) | 38.5 | 38.7 | 39.1 | 38.7 |
| Acute myocardial infarction (CC 81) | 3.7 | 3.8 | 3.9 | 3.8 |
| Other acute/subacute forms of ischemic heart disease (CC 82) | 5.9 | 5.7 | 5.8 | 5.8 |
| Chronic atherosclerosis (CC 83, 84) | 47.0 | 48.4 | 50.0 | 48.4 |
| Cardio-respiratory failure and shock (CC 79) | 18.7 | 19.7 | 21.3 | 19.9 |
| Hypertension (CC 89, 91) | 83.2 | 85.2 | 87.0 | 85.1 |
| Stroke (CC 95, 96) | 9.8 | 9.6 | 9.3 | 9.6 |
| Cerebrovascular disease (CC 97-99, 103) | 21.3 | 21.5 | 22.0 | 21.6 |
| Renal failure (CC 131) | 25.1 | 27.1 | 29.2 | 27.1 |
| COPD (CC 108) | 53.5 | 54.1 | 55.1 | 54.2 |
| Pneumonia (CC 111-113) | 41.9 | 42.1 | 42.0 | 42.0 |
| Protein-calorie malnutrition (CC 21) | 11.7 | 12.5 | 13.2 | 12.5 |
| Dementia and senility(CC 49, 50) | 28.7 | 30.6 | 31.5 | 30.2 |
| Hemiplegia, paraplegia, paralysis, functional disability (CC 67-69, 100-102,177, 178) | 8.3 | 8.5 | 8.9 | 8.5 |
| Peripheral vascular disease (CC 104, 105) | 29.6 | 30.3 | 31.6 | 30.5 |
| Metastatic cancer, acute leukemia, and other severe cancers (CC 7,8) | 9.3 | 9.4 | 9.7 | 9.5 |
| Trauma in last year (CC 154-156, 158-162) | 38.6 | 40.1 | 40.7 | 39.8 |
| Major psychiatric disorders (CC 54-56) | 12.6 | 13.3 | 14.0 | 13.3 |
| Chronic liver disease (CC 25-27) | 1.6 | 1.8 | 2.0 | 1.8 |
| Severe hematological disorders (CC 44) | 4.4 | 4.5 | 3.6 | 4.2 |
| Iron deficiency/anemias/blood disease (CC 47) | 51.4 | 54.9 | 58.9 | 55.0 |
| Depression (CC 58) | 17.5 | 20.8 | 24.3 | 20.8 |
| Parkinson’s/Huntington’s diseases (CC 73) | 4.1 | 4.2 | 4.1 | 4.1 |
| Seizure disorders and convulsions (CC 74) | 5.3 | 5.6 | 5.8 | 5.5 |
| Fibrosis of lung and other chronic lung disorders (CC 109) | 16.1 | 16.1 | 15.8 | 16.0 |
| Asthma (CC 110) | 10.8 | 11.0 | 11.4 | 11.1 |
| Vertebral fractures (CC 157) | 5.0 | 5.0 | 5.1 | 5.1 |

Table A5– Frequency of AMI Readmission Model Variables over Different Time Periods

| **Variable** | **07/2009-06/2010** | **07/2010-06/2011** | **07/2011-06/2012** | **07/2009-06/2012** |
| --- | --- | --- | --- | --- |
| Total N | 177,031 | 176,196 | 160,104 | 513,331 |
| Observed readmission rate (%) | 18.6 | 18.5 | 17.8 | 18.3 |
| Mean age minus 65 (SD) | 13.9 (8.2) | 13.9 (8.3) | 13.8 (8.3) | 13.9 (8.3) |
| Male (%) | 51.1 | 51.2 | 52.2 | 51.5 |
| History of PTCA | 9.1 | 13.1 | 17.3 | 13.0 |
| History of CABG | 5.8 | 9.0 | 12.0 | 8.8 |
| Congestive heart failure (CC 80) | 32.9 | 33.0 | 32.9 | 32.9 |
| Acute coronary syndrome (CC 81-82) | 22.5 | 22.8 | 22.6 | 22.7 |
| Anterior myocardial infarction (ICD-9 codes 410.00-410.19) | 8.0 | 7.6 | 7.2 | 7.6 |
| Other location myocardial infarction (ICD-9 codes 410.20-410.69) | 11.7 | 11.3 | 11.1 | 11.4 |
| Angina pectoris, old MI (CC 83) | 22.7 | 25.2 | 28.4 | 25.4 |
| Coronary atherosclerosis (CC 84) | 80.7 | 83.6 | 86.5 | 83.5 |
| Valvular or rheumatic heart disease (CC 86) | 26.9 | 29.4 | 32.2 | 29.4 |
| Specified arrhythmias (CC 92-93) | 34.3 | 34.9 | 35.8 | 35.0 |
| History of infection (CC 1, 3-6) | 26.6 | 26.7 | 27.1 | 26.8 |
| Metastatic cancer or acute leukemia (CC 7) | 2.1 | 2.0 | 2.1 | 2.1 |
| Cancer (CC 8-12) | 18.9 | 19.0 | 19.5 | 19.1 |
| Diabetes mellitus (DM) or DM complications (CC 15-20, 119-120) | 44.1 | 45.7 | 47.1 | 45.6 |
| Protein-calorie malnutrition (CC 21) | 5.2 | 5.9 | 6.4 | 5.8 |
| Disorders of fluid, electrolyte, acid-base (CC 22-23) | 25.9 | 27.5 | 28.9 | 27.4 |
| Iron deficiency or other anemias and blood disease (CC 47) | 40.5 | 44.9 | 48.6 | 44.5 |
| Dementia or other specified brain disorders (CC 49-50) | 17.7 | 19.0 | 20.2 | 18.9 |
| Hemiplegia, paraplegia, paralysis, functional disability  (CC 67-69, 100-102, 177-178) | 6.0 | 6.1 | 6.6 | 6.2 |
| Stroke (CC 95-96) | 7.8 | 7.6 | 7.6 | 7.7 |
| Cerebrovascular disease (CC 97-99, 103) | 20.1 | 20.7 | 21.5 | 20.7 |
| Vascular or circulatory disease (CC 104-106) | 35.7 | 36.0 | 37.0 | 36.2 |
| Chronic obstructive pulmonary disease (CC 108) | 28.8 | 30.1 | 31.5 | 30.1 |
| Asthma (CC 110) | 6.0 | 6.3 | 6.8 | 6.3 |
| Pneumonia (CC 111-113) | 23.4 | 23.6 | 23.4 | 23.5 |
| End stage renal disease or dialysis (CC 129-130) | 2.5 | 2.9 | 3.2 | 2.9 |
| Renal failure (CC 131) | 23.9 | 25.6 | 27.4 | 25.6 |
| Other urinary tract disorders (CC 136) | 20.0 | 21.0 | 22.8 | 21.2 |
| Decubitus ulcer or chronic skin ulcer (CC 148-149) | 7.8 | 7.9 | 8.1 | 7.9 |

Table A6– Frequency of HF Readmission Model Variables over Different Time Periods

| **Variable** | **07/2009-06/2010** | **07/2010-06/2011** | **07/2011-06/2012** | **07/2009-06/2012** |
| --- | --- | --- | --- | --- |
| Total N | 447,352 | 434,082 | 381,392 | 1,262,826 |
| Observed readmission rate (%) | 23.4 | 23.2 | 22.5 | 23.1 |
| Mean Age minus 65 (SD) | 15.8 (8.1) | 15.8 (8.2) | 15.9 (8.2) | 15.8 (8.2) |
| Male (%) | 45.4 | 45.5 | 46.1 | 45.7 |
| History of CABG | 9.1 | 14.7 | 19.7 | 14.2 |
| Cardio-respiratory failure or shock (CC 79) | 23.8 | 25.2 | 27.0 | 25.3 |
| Congestive heart failure (CC 80) | 76.7 | 76.9 | 77.2 | 77.0 |
| Acute coronary syndrome (CC 81-82) | 17.5 | 17.2 | 17.2 | 17.3 |
| Coronary atherosclerosis or angina (CC 83-84) | 72.4 | 73.6 | 75.3 | 73.7 |
| Valvular or rheumatic heart disease (CC 86) | 46.6 | 49.5 | 53.7 | 49.7 |
| Specified arrhythmias (CC 92-93) | 65.4 | 66.6 | 68.5 | 66.8 |
| Other or unspecified heart disease (CC 94) | 31.7 | 31.8 | 33.7 | 32.3 |
| Vascular or circulatory disease (CC 104-106) | 50.6 | 51.5 | 54.0 | 51.9 |
| Metastatic cancer or acute leukemia (CC 7) | 2.2 | 2.2 | 2.2 | 2.2 |
| Cancer (CC 8-12) | 20.8 | 20.9 | 21.6 | 21.1 |
| Diabetes or DM complications (CC 15-20, 119-120) | 52.4 | 53.5 | 54.9 | 53.5 |
| Protein-calorie malnutrition (CC 21) | 8.1 | 8.9 | 9.9 | 8.9 |
| Disorders of fluid, electrolyte, acid-base (CC 22-23) | 44.4 | 46.5 | 49.9 | 46.8 |
| Liver or biliary disease (CC 25-30) | 9.1 | 9.8 | 11.2 | 9.9 |
| Peptic ulcer, hemorrhage, other specified gastrointestinal disorders (CC 34) | 15.5 | 15.2 | 15.8 | 15.5 |
| Other gastrointestinal disorders (CC 36) | 54.3 | 58.1 | 63.7 | 58.4 |
| Severe hematological disorders (CC 44) | 4.3 | 4.5 | 3.9 | 4.2 |
| Iron deficiency or other anemias and blood disease (CC 47) | 55.3 | 59.5 | 64.4 | 59.5 |
| Dementia or other specified brain disorders (CC 49-50) | 21.1 | 22.8 | 24.8 | 22.8 |
| Drug/alcohol abuse/dependence/psychosis (CC 51-53) | 9.3 | 10.8 | 13.5 | 11.1 |
| Major psychiatric disorders (CC 54-56) | 9.5 | 10.1 | 10.8 | 10.1 |
| Depression (CC 58) | 13.5 | 16.6 | 21.1 | 16.9 |
| Other psychiatric disorders (CC 60) | 10.9 | 12.9 | 17.2 | 13.5 |
| Hemiplegia, paraplegia, paralysis, functional disability  (CC 67-69, 100-102, 177-178) | 7.4 | 7.9 | 8.8 | 8.0 |
| Stroke (CC 95-96) | 10.0 | 9.8 | 9.7 | 9.9 |
| Chronic obstructive pulmonary disease (CC 108) | 46.5 | 47.9 | 49.6 | 47.9 |
| Fibrosis of lung or other chronic lung disorders (CC 109) | 11.9 | 12.1 | 11.7 | 11.9 |
| Asthma (CC 110) | 8.7 | 9.1 | 9.7 | 9.2 |
| Pneumonia (CC 111-113) | 44.0 | 44.6 | 45.4 | 44.6 |
| End stage renal disease or dialysis (CC 129-130) | 3.8 | 4.2 | 4.8 | 4.2 |
| Renal failure (CC 131) | 45.2 | 47.8 | 50.9 | 47.8 |
| Nephritis (CC 132) | 2.8 | 3.0 | 4.1 | 3.3 |
| Other urinary tract disorders (CC 136) | 30.7 | 31.7 | 34.0 | 32.0 |
| Decubitus ulcer or chronic skin ulcer (CC 148-149) | 13.7 | 14.2 | 14.8 | 14.2 |

Table A7– Frequency of Pneumonia Readmission Model Variables over Different Time Periods

| **Variable** | **07/2009-06/2010** | **07/2010-06/2011** | **07/2011-06/2012** | **07/2009-06/2012** |
| --- | --- | --- | --- | --- |
| Total N | 371,003 | 382,700 | 336,055 | 1,089,758 |
| Observed readmission rate (%) | 17.7 | 17.7 | 17.4 | 17.6 |
| Mean Age minus 65 (SD) | 15.1 (8.2) | 15.3 (8.3) | 15.3 (8.3) | 15.2 (8.3) |
| Male (%) | 46.3 | 46.4 | 46.8 | 46.5 |
| History of CABG | 4.3 | 7.2 | 9.3 | 6.9 |
| History of infection (CC 1, 3-6) | 37.3 | 37.9 | 38.8 | 38.0 |
| Septicemia/shock (CC 2) | 7.5 | 7.9 | 8.4 | 7.9 |
| Metastatic cancer or acute leukemia (CC 7) | 4.9 | 4.9 | 5.3 | 5.0 |
| Lung or other severe cancers (CC 8) | 6.9 | 6.9 | 7.2 | 7.0 |
| Other major cancers (CC 9-10) | 17.6 | 17.6 | 18.1 | 17.8 |
| Diabetes mellitus (DM) or DM complications (CC 15-20, 119-120) | 40.3 | 41.2 | 42.3 | 41.2 |
| Protein-calorie malnutrition (CC 21) | 11.3 | 12.1 | 12.8 | 12.1 |
| Disorders of fluid, electrolyte, acid-base (CC 22-23) | 37.3 | 38.8 | 41.1 | 39.0 |
| Other gastrointestinal disorders (CC 36) | 58.5 | 61.9 | 66.0 | 62.0 |
| Severe hematological disorders (CC 44) | 4.3 | 4.4 | 3.6 | 4.1 |
| Iron deficiency or other anemias and blood disease (CC 47) | 51.6 | 54.9 | 58.7 | 55.0 |
| Dementia or other specified brain disorders (CC 49-50) | 28.5 | 30.2 | 31.3 | 30.0 |
| Drug/alcohol abuse/dependence/psychosis (CC 51-53) | 12.5 | 14.1 | 16.4 | 14.3 |
| Major psychiatric disorders (CC 54-56) | 12.8 | 13.4 | 14.1 | 13.4 |
| Other psychiatric disorders (CC 60) | 13.2 | 15.3 | 19.4 | 15.8 |
| Hemiplegia, paraplegia, paralysis, functional disability (CC 67-69, 100-102, 177-178) | 8.2 | 8.5 | 9.0 | 8.5 |
| Cardio-respiratory failure or shock (CC 79) | 19.5 | 20.5 | 22.1 | 20.6 |
| Congestive heart failure (CC 80) | 38.9 | 39.0 | 39.5 | 39.1 |
| Acute coronary syndrome (CC 81-82) | 7.7 | 7.5 | 7.6 | 7.6 |
| Coronary atherosclerosis or angina (CC 83-84) | 47.5 | 48.7 | 50.3 | 48.8 |
| Valvular or rheumatic heart disease (CC 86) | 22.0 | 23.3 | 25.3 | 23.5 |
| Specified arrhythmias (CC 92-93) | 41.3 | 42.4 | 44.3 | 42.6 |
| Stroke (CC 95-96) | 9.7 | 9.5 | 9.3 | 9.5 |
| Vascular or circulatory disease (CC 104-106) | 41.1 | 41.9 | 43.3 | 42.0 |
| Chronic obstructive pulmonary disease (CC 108) | 54.6 | 55.0 | 55.8 | 55.1 |
| Fibrosis of lung or other chronic lung disorders (CC 109) | 16.6 | 16.5 | 16.2 | 16.5 |
| Asthma (CC 110) | 11.2 | 11.3 | 11.6 | 11.4 |
| Pneumonia (CC 111-113) | 43.5 | 43.7 | 43.6 | 43.6 |
| Pleural effusion/pneumothorax (CC 114) | 15.3 | 15.9 | 16.8 | 16.0 |
| Other lung disorders (CC 115) | 46.8 | 47.1 | 46.8 | 46.9 |
| End stage renal disease or dialysis (CC 129-130) | 2.5 | 2.8 | 3.2 | 2.8 |
| Renal failure (CC 131) | 25.3 | 27.2 | 29.3 | 27.2 |
| Urinary tract infection (CC 135) | 28.5 | 28.7 | 28.7 | 28.6 |
| Other urinary tract disorders (CC 136) | 23.1 | 23.9 | 25.5 | 24.1 |
| Decubitus ulcer or chronic skin ulcer (CC 148-149) | 10.9 | 11.2 | 11.5 | 11.2 |
| Vertebral fractures (CC 157) | 5.0 | 5.0 | 5.1 | 5.0 |
| Other injuries (CC 162) | 36.3 | 38.3 | 39.0 | 37.9 |

Figure A2. Trend in Median (Interquartile Range) Hospital-Level Risk-Standardized Mortality Rates (RSMRs) over the Period between July 2009 and June 2012.

**
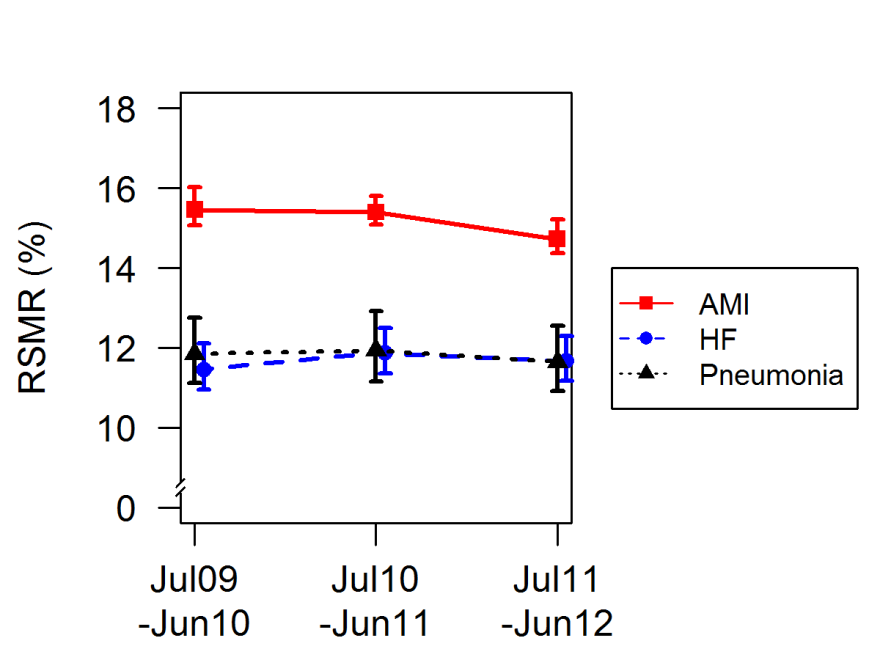
**

Figure A3. Trend in Median (Interquartile Range) Hospital-Level Risk-Standardized Readmission Rates (RSRRs) over the Period between July 2009 and June 2012.

**
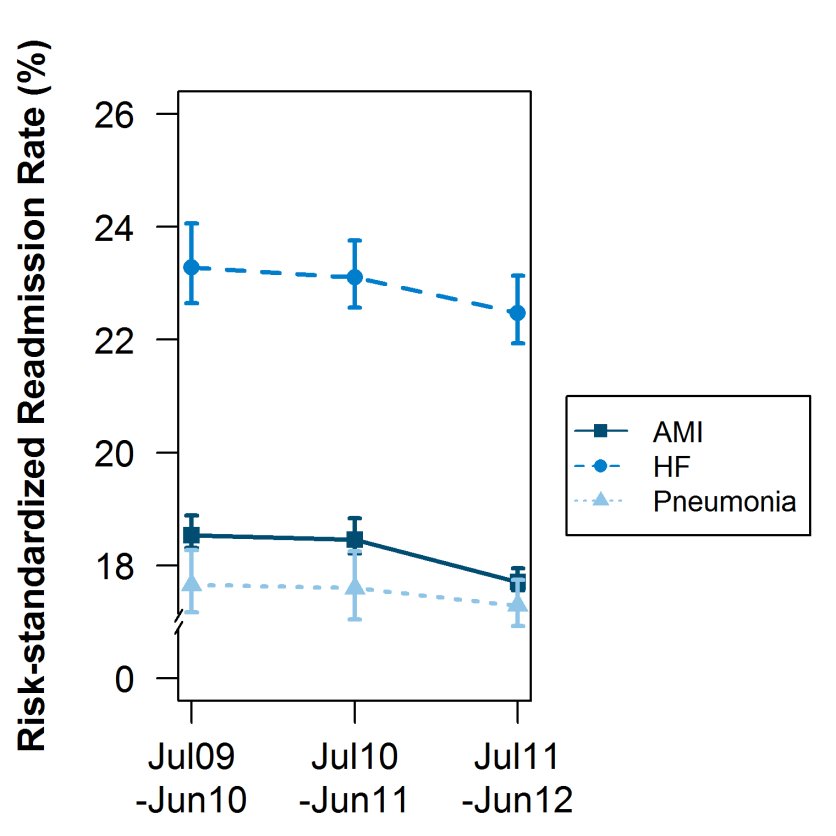
**


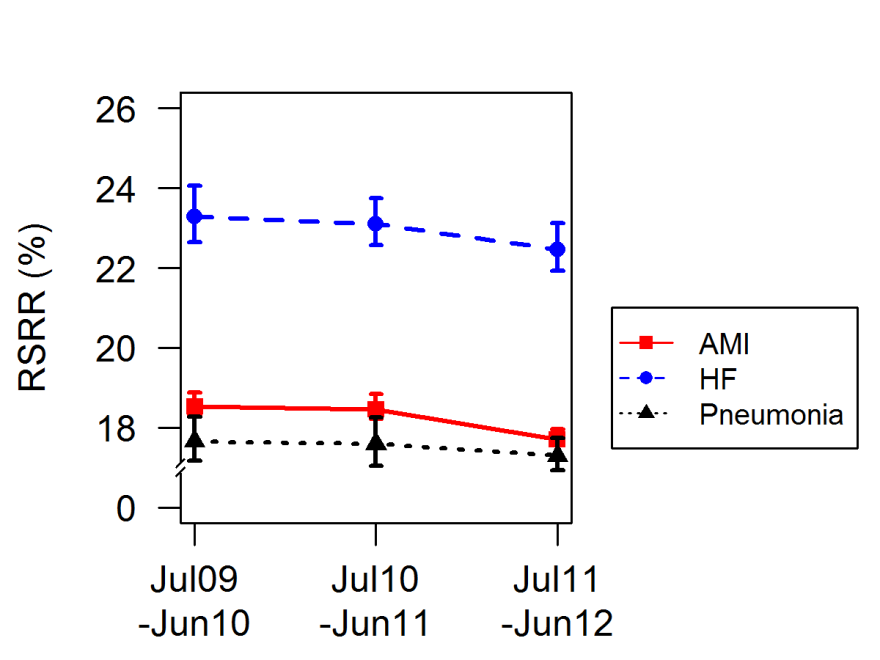


**References**

**1.** Grady J, Lin Z, Wang Y, et al. 2013 Measures Updates and Specifications: Acute Myocardial Infarction, Heart Failure, and Pneumonia 30-Day Risk-Standardized Mortality Measure (Version 7.0). 2013.

**2.** Grady J, Lin Z, Wang C, et al. 2013 Measures Updates and Specifications Report: Hospital-Level 30-Day Risk-Standardized Readmission Measures for Acute Myocardial Infarction, Heart Failure, and Pneumonia (Version 6.0). 2013.

**3.** Krumholz H, Normand SL, Galusha D, et al. Risk-Adjustment Models for AMI and HF 30-Day Mortality: Report prepared for the Centers for Medicare & Medicaid Services. 2005.

**4.** Krumholz H, Normand SL, Galusha D, et al. Risk-Adjustment Methodology for Hospital Monitoring/Surveillance and Public Reporting Supplement #1: 30-Day Mortality Model for Pneumonia: Report prepared for the Centers for Medicare & Medicaid Services. 2006.

**5.** Bhat K, Drye E, Krumholz H, et al. Acute Myocardial Infarction, Heart Failure, and Pneumonia Mortality Measures Maintenance Technical Report. 2008.

**6.** Krumholz H, Normand SL, Keenan P, et al. Hospital 30-Day Pneumonia Readmission Measure Methodology:Report prepared for the Centers for Medicare & Medicaid Services. 2008.

**7.** Krumholz H, Normand SL, Keenan P, et al. Hospital 30-Day Acute Myocardial Infarction Readmission Measure Methodology: Report prepared for the Centers for Medicare & Medicaid Services. 2008.

**8.** Krumholz H, Normand SL, Keenan P, et al. Hospital 30-Day Heart Failure Readmission Measure Methodology. Report prepared for the Centers for Medicare & Medicaid Services. 2008.

**9.** Desai M, Lin Z, Schreiner G, et al. Measures Maintenance Technical Report: Acute Myocardial Infarction, Heart Failure, and Pneumonia 30 Day Risk Standardized Readmission Measures. 2009.

**10.** Grosso L, Schreiner G, Wang Y, et al. Measures Maintenance Technical Report: Acute Myocardial Infarction. Heart Failure, and Pneumonia 30-day Risk Standardized Mortality Measures. 2009.

**11.** Bernheim S, Lin Z, Bhat K, et al. Measures Maintenance Technical Report: Acute Myocardial Infarction, Heart Failure, and Pneumonia 30 Day Risk Standardized Readmission Measures. 2010.

**12.** Bernheim S, Wang Y, Bhat K, et al. Measures Maintenance Technical Report: Acute Myocardial Infarction. Heart Failure, and Pneumonia 30-day Risk Standardized Mortality Measures. 2010.

**13.** Bernheim S, Lin Z, Grady J, et al. Measures Maintenance Technical Report: Acute Myocardial Infarction, Heart Failure, and Pneumonia 30 Day Risk Standardized Readmission Measures. 2011.

**14.** Bernheim S, Wang Y, Grady J, et al. Measures Maintenance Technical Report: Acute Myocardial Infarction. Heart Failure, and Pneumonia 30-day Risk Standardized Mortality Measures. 2011.

**15.** Bernheim S, Grady J, Spivack S, et al. 2012 Measures Maintenance Technical Report: Acute Myocardial Infarction, Heart Failure, and Pneumonia 30-Day Risk-Standardized Readmission Measure. 2012.

**16.** Bernheim S, Wang Y, Grady J, et al. 2012 Measures Maintenance Technical Report: Acute Myocardial Infarction, Heart Failure, and Pneumonia 30-Day Risk-Standardized Mortality Measure. 2012.

**17.** Horwitz L, Partovian C, Lin Z, et al. Centers for Medicare & Medicaid Services Planned Readmission Algorithm -- Version 2.1. 2013.
